# Oral Hypofunction and Risk of Weight Change among Independent Older Adults

**DOI:** 10.3390/nu15204370

**Published:** 2023-10-15

**Authors:** Chihiro Shiota, Taro Kusama, Kenji Takeuchi, Sakura Kiuchi, Ken Osaka

**Affiliations:** 1 Department of International and Community Oral Health, Tohoku University Graduate School of Dentistry, Sendai 980-8575, Japan; chihiro.shiota.s6@dc.tohoku.ac.jp (C.S.); taro.kusama.a2@tohoku.ac.jp (T.K.);; 2 Division of Statistics and Data Science, Liaison Center for Innovative Dentistry, Tohoku University Graduate School of Dentistry, Sendai 980-8575, Japan; 3 Frontier Research Institute for Interdisciplinary Sciences, Tohoku University, Sendai 980-8578, Japan

**Keywords:** mastication, cohort studies, nutrition, gerontology, oral-systemic disease

## Abstract

Oral health is essential for nutritional status; however, little is known about its association with weight change. This study aimed to investigate whether the risk of weight change differs according to the presence of each important component of oral hypofunction (fewer remaining teeth, low chewing efficiency, swallowing problems, and xerostomia) among independent older adults. This was a three-year follow-up cohort study based on self-reported questionnaires. The participants were independent older adults aged ≥65 from the Japan Gerontological Evaluation Study (JAGES). We used >5% weight loss/gain during follow-up as the outcome variables, and the number of remaining teeth (≥20/10–19/0–9), the presence of chewing difficulty, swallowing problems, and xerostomia (yes/no) as the exposure variables. We fitted the Poisson regression model, including possible confounders to estimate the risk ratios (RRs) and 95% confidence intervals (CIs). For weight loss, RRs were significantly higher among those with 0–9 remaining teeth (RR = 1.17; 95% CI = 1.11–1.23), chewing difficulty (RR = 1.12; 95% CI = 1.07–1.16), and xerostomia (RR = 1.11; 95% CI = 1.06–1.16), but there was no significant association with swallowing problems (RR = 1.01; 95% CI = 0.97–1.06). For weight gain, we also found similar associations with oral hypofunction. Oral hypofunction among older adults could have non-negligible health impacts on nutritional status.

## 1. Introduction

Malnutrition is a prevalent and critical health concern among older adults. A previous study reported that approximately a quarter of older adults are affected by malnutrition [1]. Both weight loss and gain are indicators of malnutrition among older adults [2,3]. Previous studies reveal that weight loss among older adults leads to various health problems, including cardiovascular disease, frailty, and mortality [4,5]. Weight gain is also a considerable health concern throughout life, and it is a risk factor for diabetes and various other noncommunicable diseases [6]. Therefore, the prevention of weight change among older adults is essential for preventing subsequent diseases and disorders.

The prevalence of tooth loss and other components of oral hypofunction, such as low chewing efficiency, increases with age owing to the cumulative influence of oral diseases throughout the course of life, including dental caries and periodontal disease [7,8]. Therefore, oral hypofunction is a non-negligible health condition among older adults. Previous cohort studies have reported an association between tooth loss and weight loss [9]. In addition, a previous study also reported an association between oral-health-related quality of life (OHRQoL) and body mass index [10]. However, OHRQoL is difficult to intervene and improve directly. On the other hand, oral hypofunction, such as chewing efficiency, swallowing problems, or xerostomia, could be recovered by appropriate dental treatments, including prosthodontics and oral rehabilitation. Oral hypofunction could also affect weight change; however, to our knowledge, no study has evaluated this association. In addition, weight change is bidirectional, i.e., including weight loss and gain. However, most studies have only focused on unidirectional weight change, and few studies have evaluated both weight loss and gain simultaneously.

In the present study, we hypothesized that different components of oral hypofunction affect the risk of weight change, including weight loss and gain, among older adults. However, the magnitude of the association with either direction of weight change likely differs according to the components of oral hypofunction. Therefore, the present study aimed to evaluate the association between the four components of oral hypofunction (fewer remaining teeth, low chewing efficiency, swallowing problems, and xerostomia) and weight change (weight loss/gain) among independent older adults.

## 2. Materials and Methods

### 2.1. Study Design and Participants

This was a three-year follow-up cohort study based on self-reported questionnaires. We used the data from the Japan Gerontological Evaluation Study (JAGES). The JAGES aimed to evaluate social determinants of health in cooperation with academic institutions and municipalities nationwide in Japan. The target population comprised independent older adults aged ≥65 years. They were not certified in long-term care, and their ADLs were independent. The baseline and follow-up surveys were conducted in 2016 and 2019, respectively. The survey was conducted in 28 municipalities in Japan. The questionnaires were sent to the participants and were retrieved by mail if the participants consented. We excluded the participants whose activities of daily living were not independent and those who were comorbid with dementia at baseline. In addition, we also excluded those whose information on self-reported sex, weight, and height were missing or invalid.

### 2.2. Outcome Variables

We used the incidence of >5% weight loss/gain from 2016 to 2019 as the outcome variables. Previous studies indicated that >5% weight loss is considered an indicator of an increased risk of mortality [11]. It has also been reported that both >5% weight loss and gain are associated with an increased mortality risk [4]. The validity of self-reported weight among JAGES participants was reported elsewhere [12]. The proportional weight change in percentage was calculated by dividing the weight in 2016 by that in 2019. We then categorized them as follows: “>5% weight loss”, “>5% weight gain”, and “≤5% weight change”. In the statistical analysis, we treated them as two outcomes, which were coded as “>5% weight loss” = 1 and “≤5% weight change” = 0 and “>5% weight gain” = 1 and “≤5% weight change” = 0. We also employed binary outcomes of “>5% weight loss” = 1 and ”≤5% weight change and >5% weight gain” = 0 and “>5% weight gain” = 1 and “≤5% weight change and >5% weight loss” = 0, respectively, to check the robustness for misclassification of the outcome variables [13].

### 2.3. Explanatory Variables

Fewer remaining teeth, low chewing efficiency, swallowing problems, and xerostomia are important components of oral hypofunction [14,15]. We used self-reported oral hypofunction (fewer remaining teeth, chewing difficulty, swallowing problems, and xerostomia) at baseline as the exposure variables [16]. The validity of the self-reported tooth count was reported elsewhere [17]. The questions to measure chewing difficulty, swallowing problems, and xerostomia in the present study were originally included in the Kihon checklist, which is used to assess frailty status among older Japanese adults, and the validity of these assessments was reported elsewhere [18]. For the number of remaining teeth, the question was “How many remaining teeth do you have? (Post crown and crowned teeth are included.)”, and the participants chose from among the following responses: “20 or more teeth”, “10–19 teeth”, “5–9 teeth”, “1–4 teeth”, and “0 teeth.” The answers were recategorized into “≥20 teeth”, “10–19 teeth”, and ”0–9 teeth”. For chewing difficulty, swallowing problems and xerostomia, the questions were, “Do you have any difficulties eating tough foods compared to 6 months ago?”, “Have you choked on your tea or soup recently?”, and “Do you often experience having a dry mouth?”, respectively, and if the participants answered “Yes” to each question, we defined them as having each component of oral hypofunction.

### 2.4. Covariates

The possible confounders were included as covariates based on the previous studies and clinical knowledge [19,20]. They included sex (men/women), age (65–69/70–74/75–79/80–84/≥85 years), equivalent income (JPY <2.00/2.00–3.99/≥4.0 million), education (≤9/10–12/≥13 years), comorbidities (hypertension, diabetes, cancer, and stroke), marital status (with/without a spouse), walking time as physical activity (<30/30–59/≥60 min), smoking status (never/past/current), alcohol consumption (never/past/current), use of dental prosthesis (use/nonuse), and body mass index at baseline.

### 2.5. Statistical Analysis

We used the Poisson regression model with sandwich standard error and estimated the risk ratios (RRs) and 95% confidence intervals (CIs) of the relationship between each component of oral hypofunction and the risk of >5% weight loss/gain during the follow-up [21]. We built a crude model and an adjusted model. The adjusted model included all covariates and included each oral health variable separately. For the subgroup analysis, sex- and age-stratified (men/women and <80 y/≥80 y, respectively) analyses were also conducted. Multiple imputation (MI) for missing values was conducted to reduce the selection bias [22]. We created 20 imputed datasets using multivariate imputation by chained equation (MICE) and combined the estimates obtained from each dataset based on Rubin’s rule [23]. For the sensitivity analysis, we conducted the analysis employing binary outcome and the complete case analysis. Statistical significance was set at alpha = 0.05 in all analyses. We used the Stata/MP version 17.0 for all statistical analyses (Stata Corp., College Station, TX, USA).

### 2.6. Ethical Issues

The JAGESs in 2016 and 2019 were approved by the ethics committees at Nihon Fukushi University (No. 10-05), National Center for Geriatrics and Gerontology (No.992, 1274–2), and Chiba University (No.2493, 3442). Informed consent was obtained from all participants. We followed the STROBE statement to report our observational study.

## 3. Results

**Figure 1:**
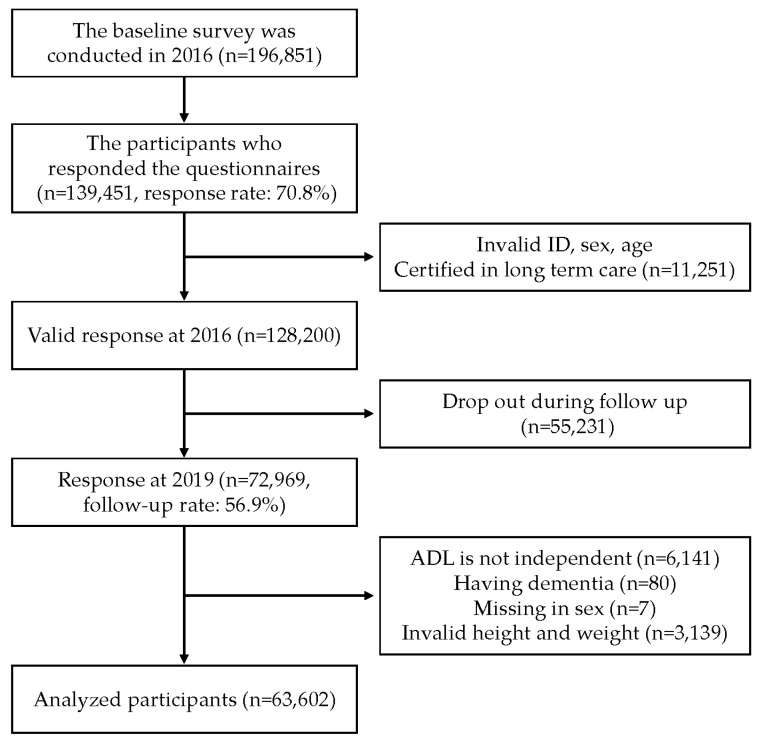
The participant flow for analytic sample.

A flow chart of participants’ inclusion is presented in Figure 1. Ultimately, we included 63,602 participants in the analysis. Supplemental Table S1 shows the characteristics of the analyzed participants at baseline before imputations. Initially, MICE imputed 15,904 participants with missing values, and Table 1 presents the characteristics of participants after the imputation. Among the analyzed participants, the mean age was 73.0 years (1SD = 5.5) at baseline, and 48.0% were men. The proportions of those who experienced >5% weight loss and gain during the three-year follow-up were 15.2% (*n* = 9676) and 10.4% (*n* = 6595), respectively. Those with ≥20, 10–19, and 0–9 remaining teeth were 60.1% (*n* = 38,210), 20.4% (*n* = 12,954), and 19.6% (*n* = 12,438), respectively. Those who had chewing difficulty, swallowing problems, and xerostomia were 23.9% (*n* = 15,181), 16.9% (*n* = 10,720), and 18.6% (*n* = 11,860), respectively. The incidence proportions of both >5% weight loss and gain were higher among those who had each component of oral hypofunction than among those who did not. Table 2 shows the result of the Poisson regression analysis. According to the result from Model 2, having fewer remaining teeth (0–9 teeth) was significantly associated with increased risk of both >5% weight loss and gain compared with those who had ≥20 remaining teeth, respectively (RR = 1.17; 95% CI = 1.11–1.23 for weight loss, RR = 1.23; 95% CI = 1.14–1.31 for weight gain). In addition, having chewing difficulty and xerostomia were also significantly associated with a higher risk of both >5% weight loss and gain, respectively (RR = 1.12; 95% CI = 1.07–1.16 for weight loss and, RR = 1.09; 95% CI = 1.04–1.15 for weight gain in chewing difficulty, and RR = 1.11; 95% CI = 1.06–1.16 for weight loss and RR = 1.09; 95% CI = 1.03–1.15 for weight gain in xerostomia). However, we did not observe the significant association between swallowing problems and both >5% weight loss and gain (*p* > 0.05). The sex- and age-stratified analyses indicated similar estimates among all participants (Supplemental Tables S2 and S3). For the sensitivity analysis, we conducted the analysis employing binary outcome and the complete case analysis, and they also suggested similar results (Supplemental Tables S4 and S5).

## 4. Discussion

We identified a positive association between oral hypofunction and the risk of weight change, indicating that those with fewer remaining teeth, chewing difficulty, and xerostomia were at significantly increased risks of both weight loss and gain. In addition, this association did not change regardless of gender or whether the person was superaged. However, we did not observe a significant association between swallowing problems and either direction of weight change. To our knowledge, our study is the first cohort study to simultaneously evaluate opposite directions of malnutrition, namely weight loss and weight gain, adding the emerging knowledge that fewer remaining teeth, low chewing efficiency, and xerostomia may be risks for both weight loss and gain among older adults to previous findings.

Our results are partially consistent with previous studies that reported tooth loss [9,20], low chewing efficiency [24,25], and xerostomia [19] were associated with increased risks of undernutrition. Our study also observed that fewer remaining teeth, chewing difficulty, and xerostomia were associated with an increased risk of weight loss, respectively. The possible mechanism for the link between fewer remaining teeth and low chewing efficiency and weight loss is considered to be as follows: Fewer remaining teeth lead to deteriorated masticatory function [26]. Subsequently, deteriorated masticatory function could cause a decrease in protein intake, such as meat [27]. Lower protein intake was reported to be associated with a higher risk of weight loss [28]; therefore, fewer remaining teeth and low chewing efficiency could increase the risk of weight loss among older adults. The association between xerostomia and weight loss can be explained as follows: as saliva is essential for mastication, xerostomia due to salivary gland hypofunction, mainly caused by polypharmacy and aging, leads to masticatory problems [28], resulting in decreased food intake, which in turn leads to undernutrition.

Conversely, we identified significant associations between fewer remaining teeth, chewing difficulty, xerostomia, and overnutrition. Previous cross-sectional studies have also reported an association between tooth loss [29], low chewing efficiency [30], and overnutrition. Another study has reported the co-occurrence of xerostomia and overnutrition [31]. A different mechanism may exist between oral hypofunction and overnutrition from that between oral hypofunction and undernutrition. Tooth loss is strongly associated with low chewing efficiency, leading to a preference for processed foods that are easier to chew, such as softer foods [32]. Additionally, xerostomia increases the duration of chewing and digestion, because dry and hard foods require more saliva for digestion [33]. Therefore, those with fewer remaining teeth, chewing difficulty, or xerostomia may have eaten foods with a high calorie content, such as processed foods containing many carbohydrates and fats, which could have led to overnutrition [34]. However, the mechanism that determines the direction of malnutrition, that is, undernutrition or overnutrition, due to oral hypofunction remains unclear. Further studies are required to investigate these underlying mechanisms.

This study did not observe a significant association between swallowing problems and weight change. No previous studies assessed the association between swallowing problems and weight gain among older adults. However, some previous cohort studies targeting older adults showed that swallowing problems were associated with the risk of weight loss [35,36,37]. In addition, other previous studies targeting community-dwelling older adults receiving home-care services reported the association of swallowing problems with undernutrition based on the Mini Nutritional Assessment—Short Form [38,39]. Although our study results are inconsistent with those of these previous studies, a possible reason for this discrepancy is that our study assessed swallowing function with a single question, whether choking occurs during fluid intake, and thus, unlike other studies, only one aspect of swallowing decline could be assessed.

From a public health perspective, oral hypofunction is a prevalent health condition affecting nutritional status among older adults [40]; therefore, prevention of prevalent oral diseases represented by dental caries and periodontal diseases throughout the life course would contribute to maintaining favorable health conditions among older adults. From a clinical perspective, deteriorated oral function can be recovered by specific interventions, which would also be essential to maintaining nutritional status among older adults. For example, oral exercise rehabilitation, such as temporomandibular joint exercises, is effective for the recovery of oral function, especially during mastication [41]. Medication management by doctors and dentists is necessary to alleviate dysphagia and dry mouth [42]. Dental prosthetic treatment also reduces the risk of weight loss [9]. However, several limitations in future dental clinical settings should be addressed regarding malnutrition due to oral hypofunction among older adults. One limitation is that other medical professionals are unfamiliar with geriatric dentistry [43]. Sharing knowledge of geriatric dentistry, including the deterioration of oral function due to aging, not only among geriatric dentists but also by other dental and medical professionals would be required [44].

There were several limitations in our study. First, regarding selection bias, there is the possibility that the participants in the present study may not have represented the targeted population; however, the response rate was 70.3%, contributing to relatively high representativeness. Although the follow-up rate of the present study was relatively low, this was mainly due to the random distribution of the questionnaire at follow-up surveys in some municipalities. This kind of dropout is considered completely at random and less likely to induce bias in the results. However, another reason for dropout in the present study was death or being certified to be eligible for long-term care. Both deteriorated oral function and malnutrition are critical risk factors for physical disability and mortality [4,45]. This could have led to underestimating the association between oral hypofunction and malnutrition in the present study. Second, we used self-reported questionnaires for information bias, which could have induced misclassification in the actual condition of the outcome or exposure. However, the validity of the question we used to measure body weight and oral health was reported previously [12]. In addition, the misclassification of outcome and exposure variables in the present study is considered to be nondifferential, and it induces bias in the result toward the null hypothesis [13]. We conducted the sensitivity analysis with the binary outcome and exposure, and we still observed similar results to the main analysis (Supplemental Table S5). Therefore, the same positive association between oral hypofunction and malnutrition would be observed even under a situation with less misclassification. In future research, an analysis with a clinical measurement of oral and nutritional status would strengthen the present results. Third, regarding confounding factors, the present study included possible confounders; however, there is the possibility of residual confounding and unmeasured confounders. 

In our study, there are several strengths. First, the present study included a large sample size, contributing to higher statistical power to detect associations. Second, this study’s participants were older Japanese adults living in multiple municipalities around Japan, contributing to the external validity of the present results. In addition, as we mentioned above, the mechanism between oral hypofunction and malnutrition is supported by previous epidemiological studies. Therefore, the findings of the present study are considered to have high generalizability. Third, the present study has a longitudinal design, contributing to eliminating the possibility of reverse causation.

## 5. Conclusions

This three-year follow-up cohort study indicates that fewer remaining teeth, low chewing efficiency, and xerostomia are associated with increased risks of >5% weight loss and gain in independent older adults. This result emphasizes the importance of maintaining favorable oral health status to prevent malnutrition among older adults.

## Figures and Tables

**Table 1:** Descriptive characteristics of the participants after multiple imputation (*n* = 63,602).

	All Participants		Weight Change during Follow-Up					
			>5% Loss		≤5% Change		>5% Gain	
	*n*	%	*n*	%	*n*	%	*n*	%
**Total**	63,602	100.0	9676	15.2	47,331	74.4	6595	10.4
**Number of remaining teeth**								
≥20 teeth	38,210	100.0	5203	13.6	29,254	76.6	3753	9.8
10–19 teeth	12,954	100.0	2173	16.8	9390	72.5	1390	10.7
0–9 teeth	12,438	100.0	2299	18.5	8687	69.8	1452	11.7
**Chewing difficulty**								
No	48,421	100.0	7021	14.5	36,534	75.5	4866	10.0
Yes	15,181	100.0	2655	17.5	10,797	71.1	1729	11.4
**Swallowing problems**								
No	52,882	100.0	7958	15.0	39,464	74.7	5460	10.3
Yes	10,720	100.0	1718	16.0	7867	73.4	1135	10.6
**Xerostomia**								
No	51,742	100.0	7627	14.7	38,862	75.1	5252	10.2
Yes	11,860	100.0	2049	17.3	8469	71.4	1343	11.3
**Sex**								
Men	30,427	100.0	4615	15.1	22,813	75.0	2999	9.9
Women	33,175	100.0	5061	15.3	24,518	73.9	3596	10.8
**Age**								
65–69	21,426	100.0	2698	12.6	16,341	76.3	2387	11.1
70–74	19,138	100.0	2635	13.8	14,510	75.8	1993	10.4
75–79	14,424	100.0	2363	16.4	10,642	73.8	1419	9.8
80–84	6604	100.0	1431	21.7	4579	69.3	594	9.0
85–	2010	100.0	549	27.3	1259	62.6	202	10.1
**Smoking status**								
Never	6395	100.0	1092	17.1	4443	69.5	860	13.5
Past	19,189	100.0	2884	15.0	14,440	75.3	1865	9.7
Current	38,018	100.0	5701	15.0	28,448	74.8	3869	10.2
**Alcohol consumption**								
Never	26,815	100.0	3754	14.0	20,446	76.2	2615	9.8
Past	6002	100.0	1020	17.0	4247	70.8	734	12.2
Current	30,785	100.0	4901	16.0	22,638	73.5	3246	10.5
**Comorbidities**								
Hypertension	27,418	100.0	4456	16.2	20,197	73.7	2765	10.1
Diabetes	7792	100.0	1566	20.1	5543	71.1	683	8.8
Cancer	2324	100.0	382	16.4	1638	70.5	304	13.1
Stroke	1404	100.0	238	16.9	997	71.1	169	12.0
**Marital status**								
Without a spouse	15,050	100.0	2422	16.1	10,910	72.5	1718	11.4
With a spouse	48,552	100.0	7254	14.9	36,421	75.0	4877	10.1
**Education (year)**								
≤9	16,610	100.0	2885	17.4	11,864	71.4	1861	11.2
10–12	27,982	100.0	4111	14.7	20,957	74.9	2914	10.4
≥13	19,010	100.0	2680	14.1	14,510	76.3	1820	9.6
**Equivalent income (million JPY)**								
<2.00	28,872	100.0	4714	16.3	21,011	72.8	3146	10.9
2.00–4.00	27,036	100.0	3895	14.4	20,435	75.6	2706	10.0
>4.00	7694	100.0	1067	13.9	5884	76.5	743	9.6
**Denture use**								
No	23,793	100.0	3347	14.1	18,032	75.8	2414	10.1
Yes	39,809	100.0	6329	15.9	29,299	73.6	4181	10.5
**Walking time (min/day)**								
<30	14,677	100.0	2638	18.0	10,390	70.8	1649	11.2
30–59	23,958	100.0	3636	15.2	17,989	75.1	2333	9.7
≥60	24,967	100.0	3402	13.6	18,952	75.9	2613	10.5
	**Mean**	**SD**	**Mean**	**SD**	**Mean**	**SD**	**Mean**	**SD**
**Body mass index**	22.8	(3.0)	23.5	(3.3)	22.8	(2.9)	22.0	(3.0)

**Table 2:** Association between oral health status and >5% weight loss/gain.

	>5% Weight Loss		>5% Weight Gain	
	(vs. ≤5% Weight Change)		(vs. ≤5% Weight Change)	
	(*n* = 57,007)		(*n* = 53,926)	
	Crude model RR (95% CI)	Adjusted model RR (95% CI) ^a^	Crude model RR (95% CI)	Adjusted model RR (95% CI) ^a^
**Oral health status**				
**Number of remaining teeth**				
≥20	1.00 (Ref.)	1.00 (Ref.)	1.00 (Ref.)	1.00 (Ref.)
10–19	1.25 (1.19–1.30) ***	1.16 (1.10–1.21) ***	1.13 (1.07–1.20) ***	1.13 (1.06–1.20) ***
0–9	1.39 (1.33–1.45) ***	1.17 (1.11–1.23) ***	1.26 (1.19–1.33) ***	1.23 (1.14–1.31) ***
**Chewing difficulty**				
No	1.00 (Ref.)	1.00 (Ref.)	1.00 (Ref.)	1.00 (Ref.)
Yes	1.22 (1.18–1.27) ***	1.12 (1.07–1.16) ***	1.17 (1.12–1.24) ***	1.09 (1.04–1.15) **
**Swallowing problems**				
No	1.00 (Ref.)	1.00 (Ref.)	1.00 (Ref.)	1.00 (Ref.)
Yes	1.07 (1.02–1.12) **	1.01 (0.97–1.06)	1.04 (0.98–1.10)	1.01 (0.95–1.08)
**Xerostomia**				
No	1.00 (Ref.)	1.00 (Ref.)	1.00 (Ref.)	1.00 (Ref.)
Yes	1.19 (1.14–1.24) ***	1.11 (1.06–1.16) ***	1.15 (1.09–1.22) ***	1.09 (1.03–1.15) **

Abbreviations: RR, risk ratio; 95% CI, 95% confidence interval; Ref, reference. ^a^ Adjusted for sex, age, smoking status, alcohol consumption, comorbidities (hypertension, diabetes, cancer, stroke), marital status, education, equivalent income, denture uses, walking time, and body mass index at baseline and including each oral health variable separately. ** *p* < 0.01, *** *p* < 0.001.

## Data Availability

Data are available upon reasonable request.

## Supplementary Materials

The following supporting information can be downloaded at https://www.mdpi.com/article/10.3390/nu15204370/s1, Supplementary Table S1: Descriptive characteristics of the participants before multiple imputation (*n* = 63,602).; Supplementary Table S2: Association between oral health status and >5% weight loss/gain stratified by sex (*n* = 63,602).; Supplementary Table S3: Association between oral health status and >5% weight loss/gain stratified by <80 y/≥80 y (*n* = 63,602).; Supplementary Table S4: Association between oral health status and >5% weight loss/gain as binary outcome (*n* = 63,602).; Supplementary Table S5: Association between oral health status and >5% weight loss/gain in complete case analysis (*n* = 47,698).
