# Supplementary material for: Oral Hypofunction and Risk of Weight Change among Independent Older Adults
Source: Nutrients. 2023 Oct 15;15(20):4370. doi: 10.3390/nu15204370 (PMC10610140; doi:10.3390/nu15204370)
Supplement: Supplementary file 1 [file nutrients-15-04370-s001.zip › nutrients-2628093-supplementary.pdf]

Title: Oral Hypofunction and Risk of Weight Change among Independent Older Adults

Authors: C. Shiota, T. Kusama, K. Takeuchi, S. Kiuchi, K. Osaka

Supplementary Materials

Contents:

- Supplementary Table S1. Descriptive characteristics of the participants before multiple imputation (n = 63,602).
- Supplementary Table S2. Association between oral health status and >5% weight loss/gain stratified by sex (n = 63,602).
- Supplementary Table S3. Association between oral health status and >5% weight loss/gain stratified by <80y/≥80y (n = 63,602).
- Supplementary Table S4. Association between oral health status and >5% weight loss/gain as binary outcome (n = 63,602).
- Supplementary Table S5. Association between oral health status and >5% weight loss/gain in complete case analysis (n = 47,698).

Supplementary Table S1. Descriptive characteristics of the participants before multiple imputation (n = 63,602).

	All participants		Weight change during follow–up >5% loss		≤5% change		>5% gain	
	n	%	n	%	n	%	n	%
**Total**	63,602	100.0	9,676	15.2	47,331	74.4	6,595	10.4
**Number of remaining teeth**								
20 teeth and over	37,844	100.0	5,127	13.6	28,999	76.6	3,718	9.8
10–19 teeth	12,758	100.0	2,132	16.7	9,259	72.6	1,367	10.7
0–9 teeth	12,221	100.0	2,249	18.4	8,549	69.9	1,425	11.7
Missing	779	100.0	168	21.6	526	67.5	85	10.9
**Chewing difficulty**								
No	48,019	100.0	6,942	14.5	36,248	75.5	4,823	10.0
Yes	15,012	100.0	2,618	17.4	10,684	71.2	1,710	11.4
Missing	577	100.0	116	20.0	399	69.2	62	10.8
**Swallowing problem**								
No	52,344	100.0	7,852	15.0	39,095	74.7	5,397	10.3
Yes	10,580	100.0	1,689	15.9	7,773	73.5	1,118	10.6
Missing	678	100.0	135	19.9	463	68.3	80	11.8
**Xerostomia**								
No	51,014	100.0	7,486	14.7	38,351	75.2	5,177	10.2
Yes	11,658	100.0	2,004	17.2	8,331	71.5	132	11.4
Missing	930	100.0	186	20.0	649	69.8	95	10.2
**Sex**								
Men	30,427	100.0	4,615	15.2	22,813	75.0	2,999	9.8
Women	33,175	100.0	5,061	15.3	24,518	73.9	3,596	10.8
**Age**								
65–69	21,426	100.0	2,698	12.6	16,341	76.3	2,387	11.1
70–74	19,138	100.0	2,635	13.8	14,510	75.8	1,993	10.4
75–79	14,424	100.0	2,363	16.4	10,642	73.8	1,419	9.8
80–84	6,604	100.0	1,431	21.7	4,579	69.3	594	9.0
85–	2,010	100.0	549	27.3	1,259	62.6	202	10.1
**Smoking status**								
Never	6,337	100.0	1,081	17.1	4,401	69.4	855	13.5
Past	19,001	100.0	2,847	15.0	14,307	75.3	1,847	9.7
Current	37,500	100.0	5,593	14.9	28,085	74.9	3,822	10.2
Missing	764	100.0	155	20.3	538	70.4	71	9.3

**Alcohol consumption**								
Never	26,393	100.0	3,680	13.9	20,135	76.3	2,578	9.8
Past	5,882	100.0	999	17.0	4,159	70.7	724	12.3
Current	30,019	100.0	4,764	15.9	22,079	73.5	3,176	10.6
Missing	1,308	100.0	233	17.8	958	73.3	117	8.9
**Comorbidities**								
Hypertension	26,656	100.0	4,326	13.2	19,648	73.7	2,682	10.1
Diabetes	7,591	100.0	1,529	20.1	5,396	71.1	666	8.8
Cancer	2,256	100.0	372	16.5	1,587	70.3	297	13.2
Stroke	1,369	100.0	231	16.9	973	71.1	165	12.0
Missing	1,894	100.0	294	15.5	1,395	73.7	205	10.8
**Marital status**								
Without a spouse	14,925	100.0	2,391	16.0	10,829	72.6	1,705	11.4
With a spouse	48,259	100.0	7,188	14.9	36,226	75.1	4,845	10.0
Missing	418	100.0	97	23.2	276	66.0	45	10.8
**Education (year)**								
≤9	16,423	100.0	2,842	17.3	11,740	71.5	1,841	11.2
10–12	27,713	100.0	4,056	14.6	20,773	75.0	2,884	10.4
≥13	18,844	100.0	2,646	14.0	14,397	76.4	1,801	9.6
Missing	622	100.0	132	21.2	421	67.7	69	11.1
**Equivalent income (million JPY)**								
<2.00	23,386	100.0	3,763	16.1	17,094	73.1	2,529	10.8
2.00–4.00	22,726	100.0	3,198	14.1	17,304	76.1	2,224	9.8
>4.00	6,588	100.0	889	13.5	5,079	77.1	620	9.4
Missing	10,902	100.0	1,826	16.8	7,854	72.0	1,222	11.2
**Denture use**								
No	23,399	100.0	3,286	14.0	17,737	75.8	2,376	10.2
Yes	39,317	100.0	6,244	15.9	28,944	73.6	4,129	10.5
Missing	886	100.0	146	16.5	650	73.3	90	10.2
**Walking time (min/day)**								
<30	14,399	100.0	2,580	17.9	10,199	70.8	1,620	11.3
30–59	23,519	100.0	3,543	15.1	17,690	75.2	2,286	9.7
≥60	24,535	100.0	3,319	13.5	18,649	76.0	2,567	10.5
Missing	1,149	100.0	234	20.4	793	69.0	122	10.6
	Mean	SD	Mean	SD	Mean	SD	Mean	SD
**Body mass index**	22.8	(3.0)	23.5	(3.3)	22.8	(2.9)	22.0	(3.0)

Supplementary Table S2. Association between oral health status and >5% weight loss/gain stratified by sex (n = 63,602).

	Men (n = 30,427)		Women (n = 33,175)	
	>5% weight loss (vs. ≤5% weight change)	>5% weight gain (vs. ≤5% weight change)	>5% weight loss (vs. ≤5% weight change)	>5% weight gain (vs. ≤5% weight change)
	RR (95% CI) [a]	RR (95% CI) [a]	RR (95% CI) [a]	RR (95% CI) [a]
**Oral health status**				
**Number of remaining teeth**				
≥20	1.00 (Ref.)	1.00 (Ref.)	1.00 (Ref.)	1.00 (Ref.)
10–19	1.18 (1.10–1.27) ***	1.13 (1.05–1.21) **	1.13 (1.02–1.24) *	1.12 (1.03–1.22) **
0–9	1.20 (1.12–1.29) ***	1.13 (1.05–1.22) **	1.22 (1.11–1.34) ***	1.22 (1.11–1.34) ***
**Chewing difficulty**				
No	1.00 (Ref.)	1.00 (Ref.)	1.00 (Ref.)	1.00 (Ref.)
Yes	1.11 (1.04–1.17) **	1.12 (1.06–1.19) ***	1.07 (0.99–1.15)	1.11 (1.03–1.19) **
**Swallowing problem**				
No	1.00 (Ref.)	1.00 (Ref.)	1.00 (Ref.)	1.00 (Ref.)
Yes	1.02 (0.95–1.09)	1.01 (0.95–1.08)	1.004 (0.92–1.10)	1.02 (0.94–1.10)
**Xerostomia**				
No	1.00 (Ref.)	1.00 (Ref.)	1.00 (Ref.)	1.00 (Ref.)
Yes	1.13 (1.06–1.21) ***	1.09 (1.03–1.16) **	1.08 (0.99–1.18)	1.10 (1.02–1.19) *

Abbreviations: RR = risk ratio; CI = confidence interval; Ref = Reference.
a Adjusted for sex, age, smoking status, alcohol consumption, comorbidities (hypertension, diabetes, cancer, stroke), marital status, education, equivalent income, denture use, walking time, and body mass index at baseline and including each oral health variable separately.
*p<0.05, **p<0.01, ***p <0.001

Supplementary Table S3. Association between oral health status and >5% weight loss/gain stratified by <80y/≥80y (n = 63,602).

	**<80y (n = 54,988)**		**≥80y (n = 8,614)**	
	**>5% weight loss (vs. ≤5% weight change)**	**>5% weight gain (vs. ≤5% weight change)**	**>5% weight loss (vs. ≤5% weight change)**	**>5% weight gain (vs. ≤5% weight change)**
	RR (95% CI) [a]	RR (95% CI) [a]	RR (95% CI) [a]	RR (95% CI) [a]
**Oral health status**				
**Number of remaining teeth**				
≥20	1.00 (Ref.)	1.00 (Ref.)	1.00 (Ref.)	1.00 (Ref.)
10–19	1.17 (1.10–1.23) ***	1.17 (1.05–1.30) **	1.12 (1.05–1.20) **	1.12 (0.93–1.35)
0–9	1.23 (1.16–1.30) ***	1.13 (1.02–1.26) *	1.23 (1.15–1.32) ***	1.14 (0.96–1.36)
**Chewing difficulty**				
No	1.00 (Ref.)	1.00 (Ref.)	1.00 (Ref.)	1.00 (Ref.)
Yes	1.13 (1.08–1.19) ***	1.09 (1.002–1.18) *	1.09 (1.03–1.15) **	1.10 (0.96–1.27)
**Swallowing problem**				
No	1.00 (Ref.)	1.00 (Ref.)	1.00 (Ref.)	1.00 (Ref.)
Yes	1.03 (0.98–1.09)	0.98 (0.88–1.07)	1.03 (0.97–1.10)	0.86 (0.72–1.02)
**Xerostomia**				
No	1.00 (Ref.)	1.00 (Ref.)	1.00 (Ref.)	1.00 (Ref.)
Yes	1.13 (1.07–1.19) ***	1.08 (0.99–1.18)	1.10 (1.03–1.16) **	1.03 (0.88–1.20)

Abbreviations: RR = risk ratio; CI = confidence interval; Ref = Reference.
a Adjusted for sex, age, smoking status, alcohol consumption, comorbidities (hypertension, diabetes, cancer, stroke), marital status, education, equivalent income, denture use, walking time, and body mass index at baseline and including each oral health variable separately.
*p<0.05, **p<0.01, ***p <0.001

Supplementary Table S4. Association between oral health status and >5% weight loss/gain as a binary outcome (n = 63,602).

	>5% weight loss (vs. ≤5% weight change and >5% weight gain)	>5% weight gain (vs. ≤5% weight change and >5% weight loss)
	RR (95% CI) [a]	RR (95% CI) [a]
**Oral health status**		
**Number of remaining teeth**		
≥20	1.00 (Ref.)	1.00 (Ref.)
10–19	1.15 (1.10–1.21) ***	1.09 (1.02–1.16) **
0–9	1.16 (1.10–1.22) ***	1.18 (1.11–1.26) ***
**Chewing difficulty**		
No	1.00 (Ref.)	1.00 (Ref.)
Yes	1.09 (1.05–1.14) ***	1.09 (1.04–1.16) **
**Swallowing problem**		
No	1.00 (Ref.)	1.00 (Ref.)
Yes	1.01 (0.96–1.06)	1.02 (0.96–1.08)
**Xerostomia**		
No	1.00 (Ref.)	1.00 (Ref.)
Yes	1.09 (1.04–1.14) ***	1.09 (1.03–1.16) **

Abbreviations: RR = risk ratio; CI = confidence interval; Ref = Reference.

a Adjusted for sex, age, smoking status, alcohol consumption, comorbidities (hypertension, diabetes, cancer, stroke), marital status, education, equivalent income, denture use, walking time, and body mass index at baseline and including each oral health variable separately.

**p<0.01, ***p <0.001

Supplementary Table S5. Association between oral health status and >5% weight loss/gain in complete case analysis (n = 47,698).

	>5% weight loss (vs. ≤5% weight change and >5% weight gain) RR (95% CI) [a]	>5% weight gain (vs. ≤5% weight change and >5% weight loss) RR (95% CI) [a]
**Oral health status**		
**Number of remaining teeth**		
≥20	1.00 (Ref.)	1.00 (Ref.)
10–19	1.17 (1.11–1.24) ***	1.11 (1.03–1.20) **
0–9	1.18 (1.11–1.26) ***	1.23 (1.14–1.33) ***
**Chewing difficulty**		
No	1.00 (Ref.)	1.00 (Ref.)
Yes	1.15 (1.09–1.21) ***	1.11 (1.04–1.18) ***
**Swallowing problem**		
No	1.00 (Ref.)	1.00 (Ref.)
Yes	1.07 (1.01–1.13) *	1.04 (0.97–1.12)
**Xerostomia**		
No	1.00 (Ref.)	1.00 (Ref.)
Yes	1.14 (1.08–1.20) ***	1.10 (1.03–1.17) ***

Abbreviations: RR = risk ratio; CI = confidence interval; Ref = Reference.
a Adjusted for sex, age, smoking status, alcohol consumption, comorbidities (hypertension, diabetes, cancer, stroke), marital status, education, equivalent income, denture use, walking time, and body mass index at baseline and including each oral health status variables separately.
*p<0.05, **p<0.01, ***p <0.001
